# Suppressed prefrontal cortex oscillations associate with clinical pain in fibrodysplasia ossificans progressiva

**DOI:** 10.1186/s13023-021-01709-4

**Published:** 2021-01-30

**Authors:** Ke Peng, Keerthana Deepti Karunakaran, Robert Labadie, Miranda Veliu, Chandler Cheung, Arielle Lee, Paul B. Yu, Jaymin Upadhyay

**Affiliations:** 1 Department of Anesthesiology, Critical Care and Pain Medicine, Boston Children’s Hospital, Harvard Medical School, Boston, MA USA; 2 Département en Neuroscience, Centre de Recherche du CHUM, l’Université de Montréal, Montreal, QC Canada; 3 Division of Cardiovascular Medicine, Department of Medicine, Brigham and Women’s Hospital, Harvard Medical School, Boston, MA USA; 4 Department of Psychiatry, McLean Hospital, Harvard Medical School, Belmont, MA USA

**Keywords:** Pain, Central nervous system, Fibrodysplasia ossificans progressiva, Functional near-infrared spectroscopy

## Abstract

**Background:**

Pain is a highly prevalent symptom experienced by patients across numerous rare musculoskeletal conditions. Much remains unknown regarding the central, neurobiological processes associated with clinical pain in musculoskeletal disease states. Fibrodysplasia ossificans progressiva (FOP) is an inherited condition characterized by substantial physical disability and pain. FOP arises from mutations of the bone morphogenetic protein (BMP) receptor Activin A receptor type 1 (ACVR1) causing patients to undergo painful flare-ups as well as heterotopic ossification (HO) of skeletal muscles, tendons, ligaments, and fascia. To date, the neurobiological processes that underlie pain in FOP have rarely been investigated. We examined pain and central pain mechanism in FOP as a model primary musculoskeletal condition. Central nervous system (CNS) functional properties were investigated in FOP patients (N = 17) stratified into low (0–3; 0–10 Scale) and high (≥ 4) pain cohorts using functional near-infrared spectroscopy (fNIRS). Associations among clinical pain, mental health, and physical health were also quantified using responses derived from a battery of clinical questionnaires.

**Results:**

Resting-state fNIRS revealed suppressed power of hemodynamic activity within the slow-5 frequency sub-band (0.01–0.027 Hz) in the prefrontal cortex in high pain FOP patients, where reduced power of slow-5, prefrontal cortex oscillations exhibited robust negative correlations with pain levels. Higher clinical pain intensities were also associated with higher magnitudes of depressive symptoms.

**Conclusions:**

Our findings not only demonstrate a robust coupling among prefrontal cortex functionality and clinical pain in FOP but lays the groundwork for utilizing fNIRS to objectively monitor and central pain mechanisms in FOP and other musculoskeletal disorders.

## Introduction

Pain is a highly common driver of reduced quality of life across rare, musculoskeletal diseases [4, 21, 32]. Prominent and more accessible features of musculoskeletal disease such as skeletal deformities and lesions, or muscle atrophy and weakness garner extensive clinical and research attention, whereas the neurobiological cause(s) of pain have been less well-studied in this clinical domain. While there have been advances in understanding central abnormalities in musculoskeletal diseases [26, 50], neurological processes underlying pain in a chronic musculoskeletal state remain largely undefined. Demonstrating that musculoskeletal pathology yields or associates with central, pain-related alteration can serve as a neurobiological basis for implementing specific pharmacological or non-pharmacological interventions. A deeper understanding of the central manifestations of pain in rare musculoskeletal diseases, an area that has received minimal attention to date, may help uncover new targetable pain mechanisms present in musculoskeletal illnesses impacting broader populations.

Here, we aimed to determine whether and how clinical pain is centrally represented in fibrodysplasia ossificans progressiva (FOP; OMIM #135100) patients. FOP is an ultra-rare disease with an estimated 1:800,000–1:3,000,000 prevalence worldwide (285 cases in the United States) [2, 30, 38]. FOP is an inherited disorder that arises from missense mutations of the type I bone morphogenetic protein (BMP) receptor Activin A receptor type 1 (*ACVR1*) [43]. The mutant form of ACVR1 confers activin-A-dependent osteogenic signaling, which results in the heterotopic ossification (HO) of soft, connective tissue structures such as skeletal muscles, tendons, ligaments, and fascia [7, 13, 14], often leading to progressive immobilization and persistent pain. Prior observations indicate that the sole presence of the ACVR1 mutation is not sufficient for HO formation, but rather, exogenous events involving soft tissue injury, inflammation, or trauma are necessary to trigger the pathological ACVR1 signaling cascade and HO in FOP [42, 49].

The induction as well as the expansion of HO lesions are often preceded by soft tissue swellings termed “flare-ups” [13, 37, 46], which are characterized by local inflammation or edema, redness or warmth of skin, joint stiffness, and pain [8, 20, 36]. In the event of a flare-up, inflammatory responses alongside sustained pressure on hard or soft tissue structures are factors that can evoke high levels of pain. Interestingly, our prior analysis of data collected from a sample of approximately 100 FOP patients demonstrate that while moderate to severe pain was reported during flare-ups, sub-populations of FOP patients in a quiescent period and showing no objective signs of a flare-up also experienced an identical level of pain [34]. This finding points to the concept that pain in FOP is associated with but not entirely driven by peripheral, flare-up-related mechanisms. Additionally, FOP patients have reported experiencing recurrent severe headaches (migraine, cluster, or tension-type headaches), neuropathic pain and somatosensory abnormalities (e.g., allodynia, hyperalgesia, numbness, or tingling), which may impact or be driven by central pain processes [22, 37, 45]. Therefore, we sought to determine if modulated central mechanisms relate to clinical pain in FOP patients using resting-state, functional near-infrared spectroscopy (fNIRS). fNIRS is a non-invasive, flexible, optical neuroimaging method that quantifies the cortical hemodynamics changes based on the neuro-vascular mechanisms. fNIRS revealed for the first time a disruption in the spontaneous oscillations localized to the prefrontal cortex in FOP patients experiencing moderate to severe clinical pain.

## Materials and methods

This investigation was approved by the Boston Children’s Hospital Institutional Review Board and met the Helsinki criteria for the study of human subjects. Each participant was given a detailed overview of study procedures as well as read and provided informed written consent prior to study participation. For individuals below 18 years of age, informed consent was also obtained from their parent or legal guardian.

### Study participants

A total of 17 consecutive patients diagnosed with FOP between 7 and 61 years of age (11 females and 6 males) gave consent and were enrolled in the current study (Table 1). Of the 17 FOP patients, 13 individuals were confirmed carriers of the classic R206H mutation. It is estimated that 97% of FOP patients harbor a gain-of-function R206H mutation in *ACVR1* [43]. One FOP patient (patient 16) had a clinical and genetic variant of FOP (personal communication with Dr. Fredrick S. Kaplan at Center for Research in FOP and Related Disorders at University of Pennsylvania), while 3 individuals had not undergone genetic testing, but possessed other FOP diagnostic features such as malformation of the big toes. Following enrollment, FOP patients provided medical history, analgesic use, demographics, clinical pain intensity rating, and fNIRS data was acquired. fNIRS data was collected in multiple settings (i.e., patients’ home or community gathering) in order to accommodate patients and families. An additional clinical pain intensity rating and completion of online clinical questionnaires occurred after the fNIRS session and within a two- to three-week period. Thus, clinical pain intensity ratings were first collected at the time of fNIRS data acquisition and subsequently, during the administration of the full battery of clinical questionnaires. Clinical questionnaires were completed at a slightly later time as some patients required special equipment or software to perform online study-related task.

**Table 1:** Patient characteristics and pain profiles

Patient	Age (year)	Gender	Pain intensity* (0–10)	Pain location*	Analgesic use**	HO location	Comments
1	16	Female	0	–	–	Jaw	Pain (5–6/10) in jaw experienced in past 7 days Oxycodone used 2 weeks prior, Ice alleviates pain
2	12	Male	0	–	Naproxen	Arm, neck, shoulders & back	Pain rarely Experienced. Persistent use of naproxen
3	29	Female	4	Left foot	–	Left foot and left shoulder	Pain (1–2/10) in left shoulder experienced in past 7 days Intermittent use of ketoprofen Wheelchair use
4	28	Female	0	–	–	Right shoulder and left hip	Pain experienced ~ 2 years ago for ~ 6 months Pain may be evoked by walking
5	22	Male	3	Back, knee, & Ankle	Oxycodone & Fentanyl	Jaw, wrist, fingers, elbows, back, hips, left knee, thigh, left calf, & ankles	Pain (7/10) in past 7 days Throbbing pain reported Persistent use of oxycodone & fentanyl Wheelchair use
6	9	Male	3	Neck & back	Ibuprofen	Neck, back, shoulder, knees	Dull pain reported Intermittent use of ibuprofen
7	32	Male	1	Lower back & ankle	–	Neck, back, left thigh, & toes	Pain (4–5/10) in past 7 days Pain can be a deep ache (back) or sharp & sudden (ankle)
8	12	Female	2	Right shoulder	Acetaminophen	Neck, back, & clavicle	Pain (2/10) also experienced 2 days prior in shoulder Intermittent use of acetaminophen
9	7	Male	0	–	–	Neck & back	Pain rarely experienced in neck or back
10	30	Female	6	Lower back, back of leg, & both feet	Acetaminophen and oxycodone	Back, arms, legs, right hip, & ankles	Acetaminophen & Oxycodone taken 2 weeks prior Intermittent use of acetaminophen and oxycodone Wheelchair use
11	46	Male	3	Jaw, neck, shoulder, & hips	Oxycodone & morphine	Jaw, shoulder, & hips	Pain (5–6/10) in hips In past 7 days. Persistent use of oxycodone & morphine Wheelchair use
12	33	Female	0	–	Acetaminophen	Jaw, neck, shoulder, elbows, fingers, rib cage, spine, ankles, hips, & thighs	Pain (2–3/10) experienced In last 7 days in neck and Shoulder Pain evoked by physical activity Intermittent use of acetaminophen Wheelchair use
13	32	Female	7	Jaw & chest	–	Jaw, neck, shoulder, arms, chest, back, hips, knees, & ankles	Pain (6–7) has been Persistently present in chest and jaw No medications taken to treat pain Wheelchair use
14	31	Female	3	Right knee	Fentanyl & gabapentin	HO present throughout entire body	Pain (8/10) in right knee in past 7 days Persistent use of fentanyl & gabapentin
15	58	Female	9	Right shoulder & back	Oxycodone	HO present throughout entire body	Severe level of pain, experienced daily Intermittent use of oxycodone Wheelchair use
16	61	Female	8	Neck & back	Acetaminophen & hydrocodone	Chest, rib cage, illiac bone and spine	Persistent pain (8/10) experienced in back Pain evoked by physical activity Persistent use of acetaminophen & hydrocodone Walking cane use
17	23	Female	4	Jaw, neck, back, left lower leg	Gabapentin & celecoxib	Jaw, neck, shoulders, back, & right hip	Pain (2/10) in hamstring, back and jaw. Pain (4/10) in left calf. Both persistent Persistent use of gabapentin & celecoxib Predisone also taken

HO, Heterotopic Ossification

^*^Self-reported ongoing pain intensity and location of pain during the fNIRS study visit. Pain levels correspond to the overall pain experienced during time of fNIRS acquisition

^**^Analgesic(s) used during the fNIRS study visit

FOP patients harbored HO of varying degree (Table 1). While some individuals presented with known HO localized to a single site (e.g., craniofacial region or middle of the back), others possessed HO lesions in multiple upper and lower body regions. FOP patients also had varying levels of physical disability with some patients being completely ambulatory and required no assistance, while others used a cane or motorized wheelchair. Nine FOP patients utilized a device (i.e., motorized wheel chair or walking cane) to assist with mobilization. One FOP patient (Patient 1) was potentially in a flare-up state during time of fNIRS acquisition and all other patients were in a quiescent state. Patients did not report experiencing recent acute illnesses (e.g., fever or flu) or physical trauma.

### Clinical questionnaires

We designated a 0–3 pain rating as low pain, while ratings ≥ 4 were classified as high pain. The rationale for choosing this criterion stems from prior work noting pain interference in patients’ daily life occurring at pain levels of 4 or more [6, 44] or that analgesic treatment is frequently sought when pain intensities are higher than 3 [11]. A battery of clinical questionnaires derived from the Patient-Reported Outcomes Measurement Information System (PROMIS; http://www.healthmeasures.net) database were utilized to capture patient reported levels of (1) pain intensity (0–10 numerical rating scale), where 0 is no pain and 10 is the worst pain imaginable, (2) manifestations of pain, (3) quality of pain (i.e., sensory & affective elements), (4) physical stress, (5) physical ability, (6) psychological stress, (7) anxiety, and (8) depressive mood. Parents or guardians of pediatric FOP patients also completed the Adult Response to Children’s Symptoms (ARCS) questionnaire. The ACRS (29 items) was utilized to determine the parents’ or guardians’ behaviors in response to the child’s pain. Study participants also provided demographic data (age and gender). Each ACRS question was scored on a five-point Likert scale (Never: 0 to Always: 4). All questionnaires were administered and completed online using REDCap (https://www.project-redcap.org). It is noted that two study participants did not fully complete questionnaires and thus the cross-correlation analysis was restricted to an N = 15 FOP patient population.

### fNIRS data acquisition

**Fig. 1:**
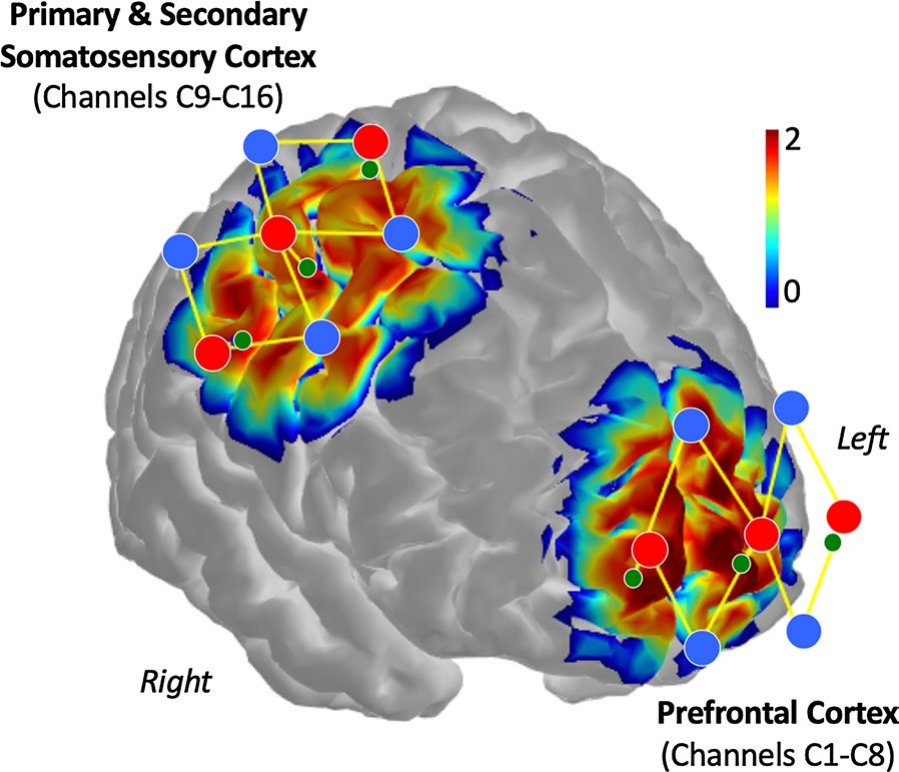
fNIRS channel arrangement. fNIRS optodes were distributed across two distinct cortical regions. A total of 6 light sources (red circles), 8 standard-separation detectors (blue circles), and 6 short-separation detectors (green circles) were utilized, where a source-detection pair (yellow lines) forms a channel. A standard source-detector distance of 30 mm was used, to yield an approximate light penetration depth of 30 mm, while short-separation detectors were positioned 8 mm from the light source to yield a shallow penetration depth. Channels C1-C8 covered primarily the bilateral medial and anterior prefrontal cortex (superior, middle, and inferior frontal gyri) and to a lesser extent, more lateral areas. Channels C9-C16 were positioned along the primary and secondary somatosensory cortex (pre- and postcentral gyri) on the right hemisphere. The sensitivity profiles for prefrontal and somatosensory channel arrangement for detecting CNS hemodynamics is represented on a logarithmic color scale (arbitrary units). Blue: 0 (minimum sensitivity); Red: 2 (maximum sensitivity)

While methods such as functional magnetic resonance imaging (fMRI) remain critical for characterizing CNS pain pathways in healthy subjects and clinical populations, undergoing an MRI-based assessment can be extremely difficult and, in some cases, impossible, due to the physical limitations experienced by FOP patients. Therefore, CNS hemodynamic properties (i.e., concentration changes of oxygenated hemoglobin (HbO), deoxygenated hemoglobin (HbR) and total hemoglobin) in FOP patients were measured using a multichannel continuous wave fNIRS system (CW7 System, TechEn, Milford, MA, USA) operating at 690 nm and 830 nm wavelengths. Each patient was first comfortably positioned either in a chair or in many cases, remained in their own wheelchair. Once study participants were positioned and at rest, pain intensity ratings (0–10 numerical rating scale) were obtained. Subsequently, an fNIRS cap—one for adults and one for children—consisting of 6 light sources, 8 standard-separation detectors, and 6 short-separation detectors was placed on the participant’s head (Fig. 1). Short-separation detectors were utilized to measure and mitigate any signal changes arising from non-cerebral sources (i.e., scalp or skull). Following the completion of fNIRS cap positioning (~ 20 to 30 min), each FOP patient was asked to remain still and visually fixate on a white cross displayed on computer screening. Resting-state fNIRS data were collected over a six-minute period.

### Data analyses

PROMIS questionnaires: For each individual patient, the summed raw scores derived from short-form, PROMIS-based questionnaires were converted to a T-score metric. The ensuing score was represented by a T-score, a standardized score with a mean ± standard deviation (SD) of 50 ± 10. Thus, a t-score of 40 is one SD below the mean. Higher scores for symptom measures (i.e., depressive symptoms) suggest worse symptom presentation, while higher scores for functional measures (i.e., cognitive function) correspond with higher functioning. Spearman's rank correlation coefficients were calculated to explore the association between each PROMIS-based measure. A false discovery rate (FDR) adjusted p-value (α = 0.05) was subsequently calculated. Statistical comparisons between low and high pain FOP study cohorts was performed using a two-tailed t-test (α = 0.05).

fNIRS data: An open-source MATLAB (Mathworks, Natick, MA, USA) toolbox, HomER2 (https://homer-fnirs.org), was utilized to preprocess all fNIRS datasets. Briefly, the raw optical intensity data were first transferred to changes in optical density by taking the logarithm of the signal. Optical density time courses were then visually inspected for quality assurance/control (QA/QC) across the 6-min acquisition period for any signal drifts or spikes, indicative of head motion (Additional file 1). During QA/QC of prefrontal cortex signals, 4 subject datasets were excluded due to excessive head motion or low signal to noise ratio, yielding 13 subject datasets for power-spectral analysis (Pain Level: 0–3; N = 7 & Pain Level: 4–10; N = 6). Separate QA/QC of somatosensory cortex signal resulted in 10 viable datasets (Pain Level: 0–3; N = 5 & Pain Level: 4–10; N = 5). Filtered (0.01–0.5 Hz) optical density time courses were further converted to concentration changes in HbO, HbR and total hemoglobin using the modified Beer-Lambert Law with a partial pathlength factor of 6. Finally, a linear regression model was employed to regress out superficial noises from the time series of each cortical fNIRS channel (i.e., having a distance of 3 cm) by including the hemoglobin time course of the short separation channel (i.e. having a distance of 8 mm) with the highest correlation as a covariate in the linear model. A constant regressor with the value of 1 was also added to the model.

Power spectral analysis of low-frequency oscillations on the recorded fNIRS hemodynamic signals was performed to investigate the changes in neuronal oscillatory dynamics with pain. Here, the HbO data were utilized rather than HbR data, based on the availability of higher signal-to-noise ratio of HbO compared to HbR time courses [23]. For each patient, the 6-min HbO time courses of each channel were first transformed to the frequency domain using the fast Fourier transform. The power of each frequency component was obtained by taking the square of its absolute amplitude value. Furthermore, prior work has shown that cortical oscillations occur in distinct frequency bands, where each range is associated with unique physiological functions [3]. Thus, we further subdivided the low-frequency oscillations range into five sub-bands: slow-5 (0.01–0.027 Hz), slow-4 (0.027–0.073 Hz), slow-3 (0.073–0.198 Hz), slow-2 (0.198–0.25 Hz), and slow-1 (0.25–0.5 Hz). For each sub-band, we conducted two-sample t-tests on the mean power values of each normal fNIRS channel between the high pain group and low pain group to test the null hypothesis that patients with high pain levels do not have a statistically significant reduction of the sub-band power of a particular channel (and therefore the underlying brain region). Channel-wise FDR correction was then applied to control the number of false positives. FDR adjusted (α = 0.05) p-values were utilized to determine significance. Resting-state functional connectivity was also explored (Additional file 1).

## Results

### Pain, mental health and physical health

**Fig. 2:**
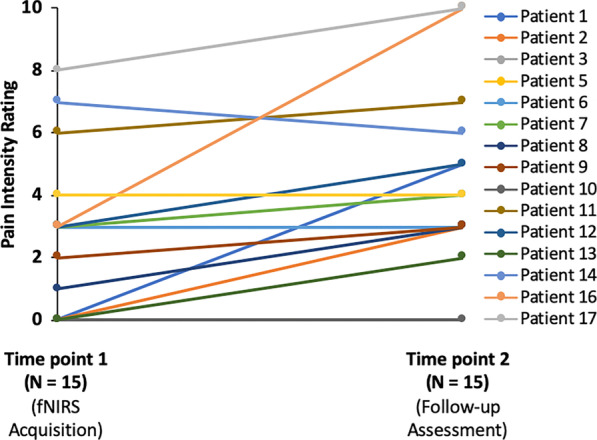
Dynamics of pain in FOP. Pain levels in FOP patients were quantified at two time points approximately 2–3 weeks apart (N = 15). Patients 4 and 15 were lost to follow-up. In some patients, pain levels were constant between the two assessments, while for others, there were increases or decreases in self-reported pain intensity. Median self-reported pain intensities were 3 and 4 for fNIRS and clinical questionnaire sessions, respectively

**Fig. 3:**
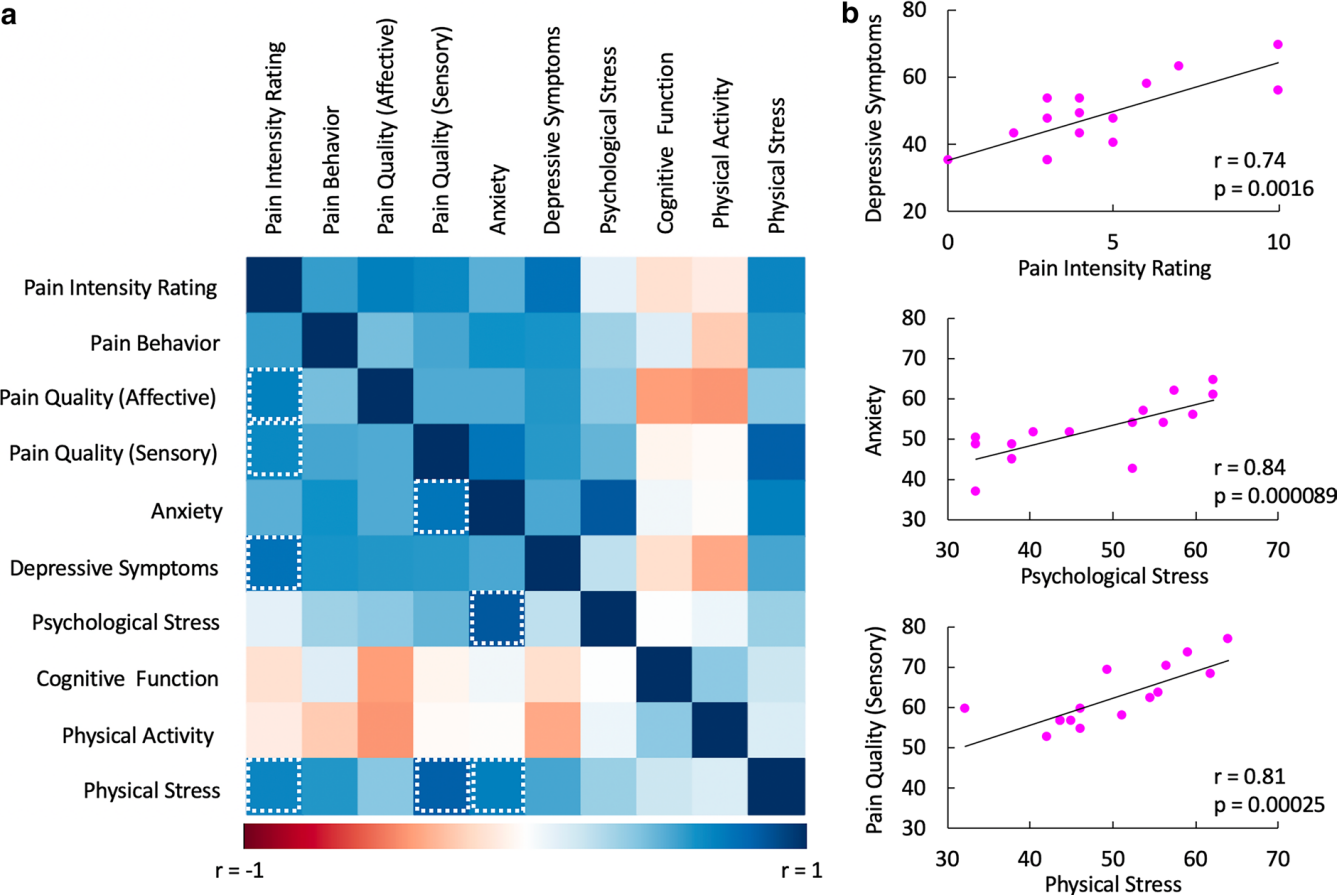
Associations among clinical pain, mental health, and physical health in FOP. **a** The cross-correlation matrix demonstrates the relationships or lack thereof between measures of pain, mental health and physical health as measured by questionnaires derived from the PROMIS database. Dotted white boxes denote significant effects surpassing multiple comparison correction (FDR-p_corr_ < 0.05). Correlation analysis was performed using data collected from 15 FOP patients. **b** Example correlation plots depicting significant relationships among measures of pain, mental health, and physical health. Within the top and bottom plots, 2 patients had overlapping data points. Data from 15 FOP patients are shown

Of the 17 enrolled FOP patients (Table 1), 11 individuals (6 males, 5 females) were categorized in the low pain (Pain Level: 0–3/10) cohort at the time of fNIRS data acquisition, while 6 female patients were allotted to the high pain (Pain Level: ≥ 4) group. In order to compare patients’ gender with low pain vs. high pain, the Fisher’s Exact test was utilized. We observed a significant association between gender and pain (*p* = 0.043). Pain ratings between participants less than 18 years of age vs. adult patients was moderately significant (*p* = 0.046). A comparison of pain levels between the first (fNIRS data acquisition) and second (clinical questionnaire completion) evaluation time points demonstrated that while pain intensity remained relatively stable for many FOP patients, others showed increases or decreases in pain (Fig. 2). Moreover, no relationship among gender and pain was present at the second clinical pain intensity assessment (*p* = 0.14). Similarly, a significant difference between FOP patients less that 18 years compared to individuals over the age of 18 was not observed at the subsequent time point (*p* = 0.18). Analgesics, including prescription opioids, were used by both study populations with some patients utilizing treatments on an intermittent or as needed basis, while others had more consistent usage. A Fisher’s Exact test was utilized to assess the association between pain (≥ 4 vs < 4) and analgesic usage (Any usage = 1 vs. Otherwise = 0). No association between pain and analgesics usage was observed with the p-value being highly non-significant (*p* = 1.0). Clinical questionnaire data acquired from FOP patients showed that individuals in the high pain cohort (N = 7) relative to the low pain group (N = 9) had altered emotional functioning in domains such as anxiety in conjunction with physical symptoms, mainly physical stress (Table 2). The interactions between pain intensity, the quality of pain (affective vs. sensory), mental health and physical health in FOP, all of which were self-reported assessments, were also characterized (Fig. 3). Of note, the intensity of pain was significantly correlated magnitude of self-reported depressive symptoms (r = 0.74, *p* = 0.0016) in addition to the magnitude physical stress experienced (r = 0.65, *p* = 0.009). Interestingly, significant relationships were further observed between level of anxiety experienced by patients, sensory aspect of pain and physical stress, suggesting an interaction among these three domains in FOP. Finally, across pediatric FOP patients, the mean ± SE ARCS questionnaire response was 2.31 ± 0.26. Low ARCS scores were likely reflective of low pain levels experienced by pediatric FOP patients.

**Table 2:** Group-level PROMIS scores

PROMIS scale	Pain: 0–3 Mean ± SE (N = 6)	Pain: 4–10 Mean ± SE (N = 9)	z-statistic	*P* value
Pain intensity rating	2.33 ± 1.21	6.11 ± 2.42	2.93	0.0033
Pain behavior	43.55 ± 11.90	53.8 ± 5.81	1.87	0.062
Pain quality (affective)	40.53 ± 4.60	49.22 ± 7.23	2.07	0.038
Pain quality (sensory)	43.20 ± 6.25	54.71 ± 6.67	2.33	0.020
Anxiety	38.82 ± 7.91	54.00 ± 8.14	2.20	0.028
depressive symptoms	41.58 ± 7.69	53.26 ± 9.35	1.80	0.072
Psychological stress	48.97 ± 6.60	54.60 ± 7.47	1.27	0.20
Cognitive function	34.95 ± 8.42	31.40 ± 6.55	0	0.38
Physical activity	34.32 ± 5.12	35.32 ± 7.12	0.20	0.84
Physical stress	56.28 ± 2.39	66.61 ± 6.69	2.47	0.014

Low (pain level: 0–3) versus high (pain level: 4–10) pain cohorts

SD, standard deviation

### Power spectral analysis (Low vs. high pain FOP patients)

**Fig. 4:**
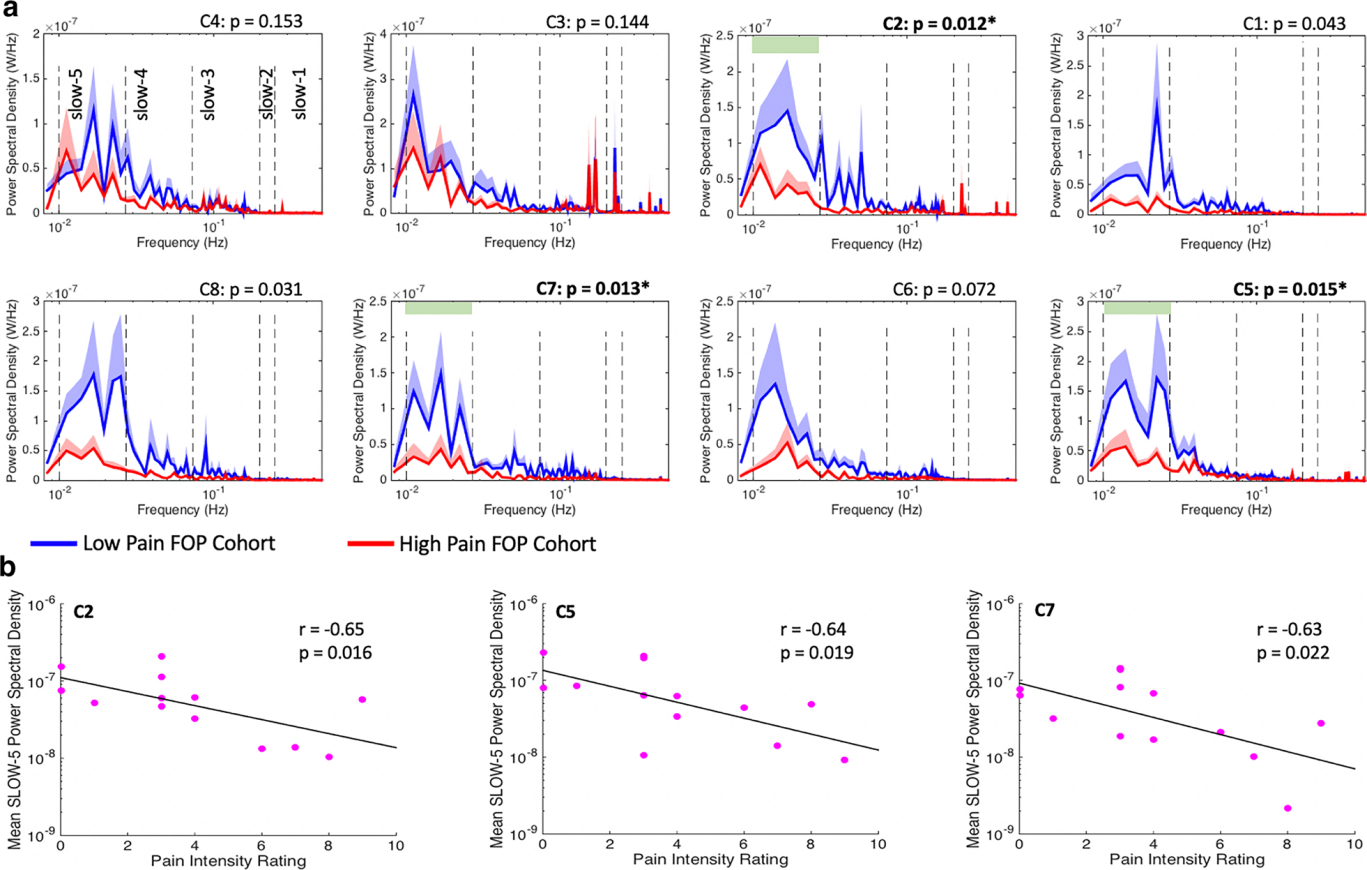
Decreased prefrontal cortex slow-5 (0.01–0.027 Hz) power in FOP patients with high pain levels. **a** Frequency analyses reveal decrease power within the slow-5 sub-band across multiple channels distributed over the medial and anterior aspects of the prefrontal cortex. A significant effect after FDR correction was observed in channels C2, C5, and C7. * denotes FDR correct p-values, while light green bars in C2, C5, and C7 plots further define the slow-5 sub-band. Mean power spectral density and standard error (shaded area) across all sub-bands (i.e., slow-1 to slow-5) are plotted for low (Pain Level: 0–3; N = 7) and high (Pain Level: 4–10; N = 6) clinical pain FOP cohorts. Clinical pain intensity levels for each patient were collected from each FOP patient just prior to acquisition of the six-minute resting-state fNIRS scan. **b** Correlation analyses performed across all FOP patients showed that for channels C2, C5, and C7, a greater extent of power suppression within the slow-5 frequency range corresponded with greater clinical pain intensity

**Fig. 5:**
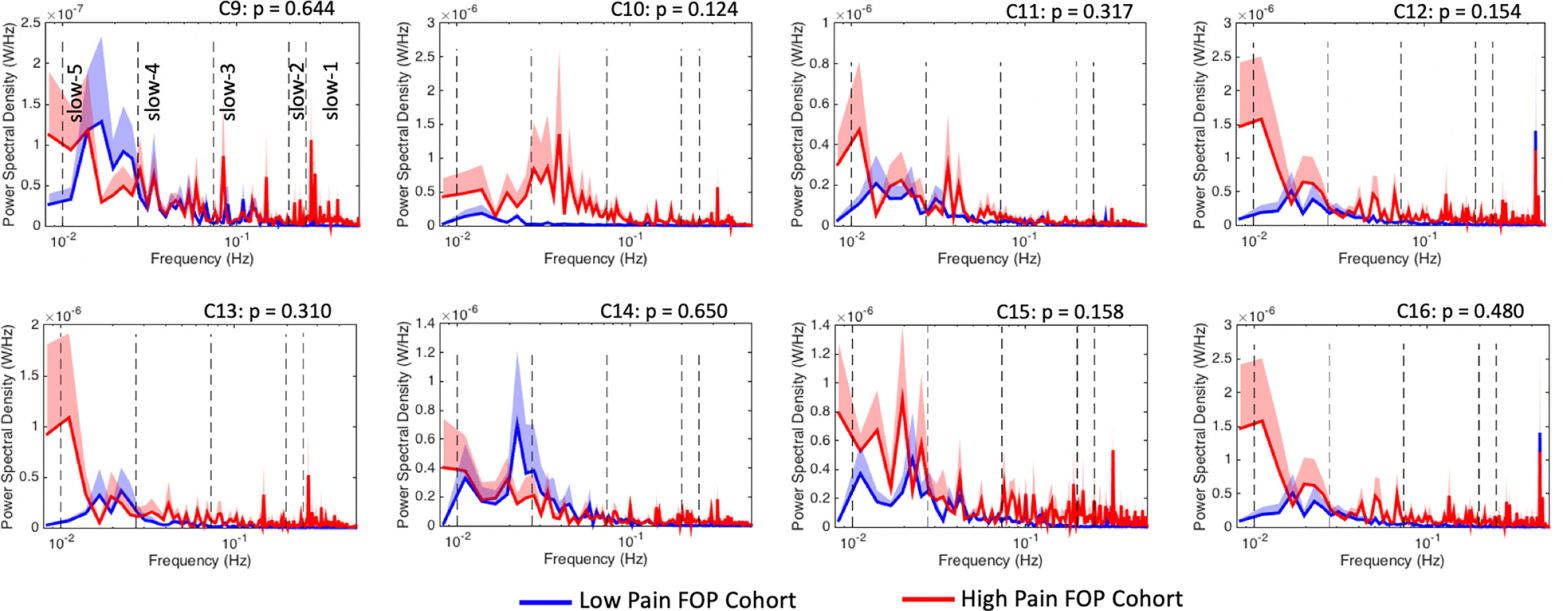
No change in primary and secondary somatosensory cortex slow-5 (0.01–0.027 Hz) power (low pain vs. high pain FOP patients). Frequency analyses reveal no significant differences in power within the slow-5 sub-band across all channels distributed over the primary and secondary somatosensory cortex. Calculation of effect sizes (Cohen’s *d* (Range): 0.03–0.79) demonstrated a low to moderate pain-dependent effect on slow-5 power in somatosensory cortices. Mean power spectral density and standard error (shaded area) across all sub-bands (i.e., slow-1 to slow-5) are plotted for low clinical pain (Pain Level: 0–3; N = 5) and high clinical pain (Pain Level: 4–10; N = 5) FOP cohorts. Clinical pain intensity levels from each FOP patient were collected just prior to acquisition of a six-minute resting-state fNIRS scan. Significant correlation was not detected between clinical pain intensity ratings and slow-5 power (Correlation (r): − 0.04 to 0.23). While a trend towards higher (relative to the low pain FOP group) power in the slow-4 sub-band (Channel C10) for the high pain FOP cohort was observed, a significant intergroup difference was not present (*p* = 0.137)

As shown in Fig. 4a, relative to the low pain cohort (N = 7), the amplitude of low-frequency oscillations localized to the prefrontal cortex and within the slow-5 (0.01–0.027 Hz) sub-band were significantly lower in high pain FOP patients (N = 6). A robust suppression of slow-5 power was observed in the high pain group across medial (channels C2 (FDR-p_corr_ = 0.012) and C7 (FDR-p_corr_ = 0.013)) as well as more lateral (channel C5 (FDR-p_corr_ = 0.015)) components of the prefrontal cortex (Cohen’s *d*: C2 = 1.51; C5 = 1.45; C7 = 1.46). Across all study participants, self-reported pain levels were negatively correlated with prefrontal cortex, slow-5 power (Fig. 4b). Pain score obtained from the second pain evaluation were also negatively correlated with the magnitude of prefrontal cortex, slow-5 power (C2: r = − 0.60, *p* = 0.039; C5: r = − 0.28, *p* = 0.39; C7: r = − 0.73, *p* = 0.007). A significant correlation was not detected between slow-5 power and severity of depressive symptoms (r = − 0.36 to − 0.41; *p* > 0.05). The age of the patient did not correlate with the magnitude of prefrontal cortex, slow-5 power (C2: r = − 0.06, *p* = 0.84; C5: r = 0.16, *p* = 0.60; C7: r = − 0.27, *p* = 0.37). As low-frequency oscillations at the slow-5 range are involved with long-range interaction of brain regions, these finding suggests that FOP patients experiencing higher levels of pain likely possess a greater magnitude of dysfunctional long-range communication involving the prefrontal cortex [3, 5, 40]. (See also resting-state functional connectivity analysis results in Additional file 1). In other words, higher self-reported pain in FOP was associated with greater disruption of large-scale CNS networks encompassing the prefrontal cortex. Figure 5 demonstrates that slow-5 amplitude alterations were not significantly different over the primary and secondary somatosensory cortices (pre- and post-central gyrus) when comparing low (N = 5) vs. high pain (N = 5) FOP patients. Calculation of effect sizes (Cohen’s ***d ***(Range): 0.03–0.79) indicated a low to moderate pain-dependent effect on slow-5 power in somatosensory cortices. A correlation between clinical pain intensity ratings and slow-5 power for any somatosensory channel, channels C9-C16, was not observed (r = − 0.04 to 0.23; FDR-adjusted *p* > 0.05). Significant differences between low and high pain FOP patients were not detected in other frequency sub-bands (i.e., slow-1 to slow-4) and in either prefrontal or somatosensory cortices.

## Discussion

This work highlights the central representation of a critical element of FOP, namely, pain. We report that the pain experienced by FOP patients was integrated with an individual’s emotional health, which points to central involvement in FOP pain. By utilizing fNIRS, we demonstrated that the severity of clinical pain was negatively associated with the power of low-frequency oscillations localized to the prefrontal cortex, a region implicated in emotional appraisal [27], reward mechanisms [29], and clinical conditions including major depressive disorder [18]. The observed loss of the prefrontal cortex, low-frequency activity within the slow-5 sub-band, strongly points to the disintegration of large-scale CNS networks in a high pain, FOP state. The current findings revealed fNIRS's utility for characterizing central pain mechanisms in FOP and suggests that this approach might be generalized for discovering central features of clinical pain in other musculoskeletal diseases.

Peripheral pathological features (nerve compression) or events (flare-ups) can cause patients to experience pain. However, across rare and also more common musculoskeletal diseases, there is a frequent discrepancy between objective measures of disease burden or peripheral trauma and patient-reported pain levels [21]. Finding such as those centered around prefrontal cortex functionality in FOP pain patients in conjunction with the elucidation of diminished emotional health may help explain this discordance between pain and tissue pathology, and better direct the implementation of pharmacological and non-pharmacological pain treatment interventions. Therefore, while the current set of results are specific to FOP, there are implications that extend beyond this rare disease and into other clinical conditions characterized by pain and musculoskeletal phenotypes.

In FOP, HO of musculoskeletal tissue causes long-term, severe disability. However, alongside the physical limitations that many FOP patients undergo and which can begin during childhood stages of life [10, 33, 36], the core pathological elements of FOP such as HO induction and growth may lead to other downstream manifestations [34]. Pain in FOP, is most often associated with recurrent soft tissue swelling or flare-ups, which in many circumstances precedes new HO lesions or expansion of existing ones [36, 37, 46]. Yet, as previous work [34] as well as the current study have revealed, clinical pain is frequently reported by FOP patients during what are considered ‘quiescent’ or non-flare-up states.

We found that pain in FOP can be variable, both across subjects and time. Monitoring patients across a 2–3-week period demonstrated intra-subject fluctuations in pain intensity with some individuals reporting increased levels of pain during this interval, while others showed little to no change in pain intensity. The site of pain often corresponded to the location of known HO lesions; however, there were cases where pain was altogether absent, or was experienced only across a subset of HO lesion sites. A pain level ≥ 4 was moderately associated with gender. A trend of higher pain levels or higher chronic pain prevalence in female FOP patients is in accord with the large body of data demonstrating higher perception of pain and more frequent occurrence of chronic pain conditions in women [31, 47]. Additionally, slightly higher pain levels were measured in older FOP patients relative to younger patients; an observation that was previously observed in other rare musculoskeletal conditions [21]. From a battery of clinical questionnaires, the perception of pain in individuals reporting a high level of pain consisted of both sensory and affective elements. Cross-correlation analyses further indicated the interaction between severity of pain, pain quality, physical health, and emotional health; however, two key features stemming from this approach were derived. First, there was clear evidence that the physical (i.e., physical stress) and psychological (i.e., depressive mood or anxiety) challenges that FOP patients harbor are tightly integrated. Second, there was a robust association between higher levels of clinical pain and greater depressive symptoms. While higher pain severity often coincides with a greater depressive symptomology in many clinical conditions [16], the observation of this relationship in a FOP population has additional implications. Pain in FOP can of course be driven by peripheral pathological events, for example, the soft tissue edema or inflammation occurring during a flare-up episode. However, superimposed upon peripheral, pain-inducing occurrences, are abnormalities anchored within the CNS, which can result in a top-down dysregulation of pain perception and relatedly, heightened pain sensitivity or maintain a persistent state of pain [17, 24].

Implementation of fNIRS enabled an objective assessment of CNS function in FOP patients and equally important, an understanding of whether and how cortical activity relates to subjective accounts of pain in this disease. Patients with moderate to severe pain were characterized by a reduced amplitude of slow-5 (0.01–0.027 Hz), low-frequency oscillations occurring within the prefrontal cortex, where higher clinical pain severity negatively correlated with more suppressed prefrontal cortex fluctuations. Using resting-state functional magnetic resonance imaging (fMRI), highly similar frequency-dependent changes have been reported in multiple chronic pain conditions. In trigeminal neuralgia [52], postherpetic neuralgia [12], and chronic back pain [28, 51] populations, a loss of slow-5 power was not only noted in prefrontal cortex [52] but also in subcortical regions such as the nucleus accumbens, which share projections with sub-regions of the frontal cortex areas amongst other (meso-)limbic structures. Thus, in general, the suppression of the frontal cortex (potentially along the frontostriatal pathway) low-frequency oscillation in the slow-5 sub-band may represent a maladaptive cortical process that is derived from persistent pain or mechanisms that sustain a pain state. Further investigation is necessary to determine if and how there is a causal link among suppressed frontal cortex oscillations and chronic pain in FOP.

The alterations in low-frequency dynamics have been previously linked with changes in functional connectivity [1]. Slow-5 oscillations are more closely associated with spontaneous neuronal oscillation intrinsic to cortical regions, including the prefrontal cortex, while higher frequency activity (e.g., slow-4 sub-band: 0.027–0.073 Hz) involves cortical and subcortical regions such as the basal ganglia [39, 48]. Slow-5 oscillations enable long-range connectivity, particularly that which is necessary for proper communication amongst CNS hubs (i.e., prefrontal cortex) embedded within large-scale networks (i.e., default-mode or mesolimbic network). A disruption of mechanisms that facilitate long-range CNS communication may underpin the decreased prefrontal, and somatosensory cortex functional connectivity observed in FOP patients with higher self-reported clinical pain levels. The somatosensory cortex encodes the sensory dimension of the pain [15], while the prefrontal cortex with its connections to the pain modulation centers (periaqueductal grey) is known to exhibit endogenous antinociceptive effects. A decrease in the connectivity of the somatosensory cortex and prefrontal cortex may imply an imbalance in the pain encoding process and the ensuing descending control of pain leading to prolonged pain status. Interestingly, relative to short-range connectivity, there is also a significantly stronger coupling between long-range connectivity and CNS metabolic properties (cerebral blood flow, cerebral metabolic rate for oxygen or glucose [25], possibly suggesting that cerebral metabolic alterations may especially perturbate slow-5 or slow-4 low-frequency oscillations and in parallel, connectivity within large-scale networks.

We project that the frequency-dependent and pain-dependent effects observed in our FOP cohort of patients are a collective and cyclical outcome of living with a chronic illness such as FOP, withstanding recurrent and severely painful flare-up episodes as well as enduring persistent pain over several months or even years. Nonetheless, the impact of aberrant activin-A- or ACVR1/ALK2-dependent activity with the CNS must be considered. In FOP patients [19, 41] lesions or focal MRI hyper-intensities along white matter pathways have been noted. Disruption of white matter integrity can indeed modulate CNS functional properties.

This investigation represents the first functional CNS imaging study in FOP and a novel application of fNIRS. fNIRS was implemented as an alternate approach to probe CNS function, as MRI-based assessments are not easily feasible for many FOP patients. This study, in conjunction with other recent efforts, demonstrates fNIRS's utility towards elucidating central pain mechanisms [9, 35]. However, limitations are noted. fNIRS provides an assessment of cortical (dys-)function, where alterations in the brainstem or subcortical structures and their association with clinical pain cannot be elucidated. Compared to fMRI, fNIRS offers flexibility in terms of implementation, allowing bedside measurement to be made. However, a low signal to noise ratio at specific optode locations, possibly due to hair, was a limiting factor. Some FOP patients presented with HO in the neck, shoulder, and upper back region, which anatomically altered their head position and caused difficultly in fNIRS positioning of optodes, particularly over the somatosensory cortices. The use of wireless fNIRS setups or implementation of advanced light source technology may facilitate improved fNIRS data quality throughout the brain. Additionally, in the current cohort of FOP patients, those categorized in the high pain group were primarily women, which is in line with prior FOP studies [22]. Nonetheless, future investigations may aim to enroll more male FOP patients with moderate to severe pain. Lastly, some patients continued analgesic treatment during the study, but the varied medication usage between patients makes this unlikely.

## Conclusions

In conclusion, fNIRS was applied to characterize cortical function in FOP patients in low and high pain states. Suppressed power within the slow-5 frequency band of prefrontal cortex in FOP patients was associated with greater clinical pain. We also have demonstrated the interaction amongst pain, mental health and physical health in FOP. This investigation provides further rationale for utilizing fNIRS to further elucidate mechanisms of pain modulation in FOP and other musculoskeletal disorders.

## Supplementary Information

**Additional file 1**. QA/QC and functional connectivity analyses of fNIRS data.

## Data Availability

The datasets generated and/or analyzed during the current study are not publicly available due institutional regulations, but are available from the corresponding author on reasonable request.

## Abbreviations

ACVR1: Activin A receptor type 1
ARCS: Adult Response to Children’s Symptoms
BMP: Bone morphogenetic protein
CNS: Central nervous system
FOP: Fibrodysplasia ossificans progressiva
FNIRS: Functional near-infrared spectroscopy
HO: Heterotopic ossification
HbO: Oxygenated hemoglobin
HbR: Deoxygenated hemoglobin
PROMIS: Patient-Reported Outcomes Measurement Information System
QA/QC: Quality assurance/quality control
