# Supplementary material for: Suppressed prefrontal cortex oscillations associate with clinical pain in fibrodysplasia ossificans progressiva
Source: Orphanet J Rare Dis. 2021 Jan 30;16:54. doi: 10.1186/s13023-021-01709-4 (PMC7847608; doi:10.1186/s13023-021-01709-4)
Supplement: Supplementary file 1 — Additional file 1. QA/QC and functional connectivity analyses of fNIRS data. [file 13023_2021_1709_MOESM1_ESM.docx]

Suppressed Prefrontal Cortex Oscillations Associate with Clinical Pain

in Fibrodysplasia Ossificans Progressiva

Ke Peng^1,2‡^, Keerthana Deepti Karunakaran^1‡^, Robert Labadie^1^, Miranda Veliu^1^, Chandler Cheung^1^, Arielle Lee^1^, Paul B. Yu^3^, Jaymin Upadhyay^1,4*^

^1^ Department of Anesthesiology, Critical Care and Pain Medicine, Boston Children’s Hospital, Harvard Medical School, Boston, MA USA.

^2^ Département en Neuroscience, Centre de Recherche du CHUM, l'Université de Montréal, Montreal, QC, Canada.

^3^ Division of Cardiovascular Medicine, Department of Medicine, Brigham and Women's Hospital, Harvard Medical School, Boston, MA 02115, USA.

^4^ Department of Psychiatry, McLean Hospital, Harvard Medical School, Belmont, MA USA.

^‡^These authors contributed equally to this work.

**Supporting Information**

**QA/QC of fNIRS Datasets**

**
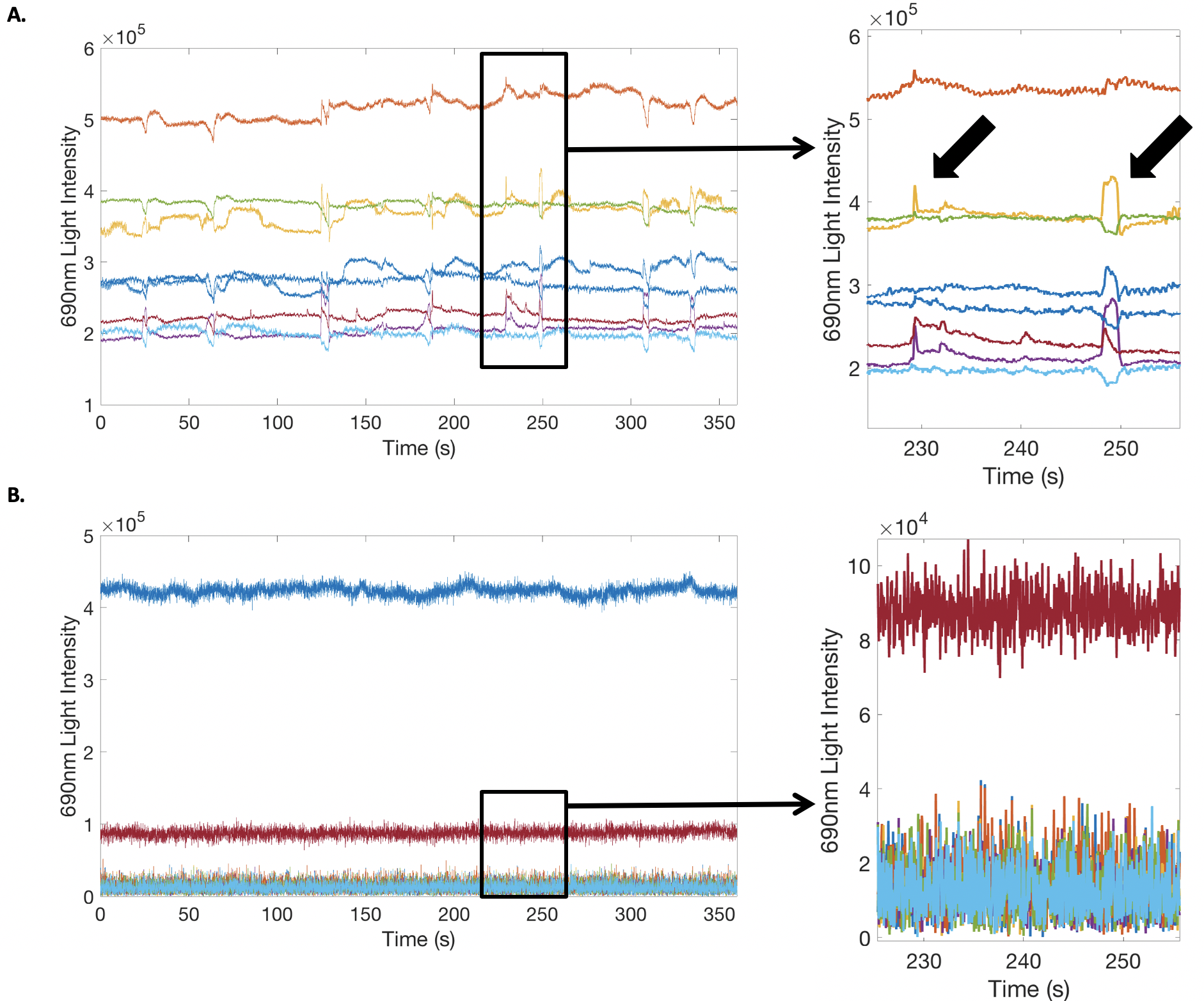
**

**Supplemental Fig. 1. QA/QC** Example datasets that were excluded from the analysis in the data quality assessment stage are shown. **A.** Prefrontal cortex raw fNIRS light intensity signals. A large number of motion artifacts (see the black arrows for examples) were noted in this particular recording session. **B.** Raw fNIRS light intensity signals from the somatosensory cortex. Poor signal-to-noise ratio from this location was noted. Oscillations due to physiological events such as heartbeats or respiration could not be seen in the data.

**Functional Connectivity Analyses of fNIRS Data**

Methods: Intrinsic, resting-state functional connectivity was also explored by first applying distinct low frequency, bandpass filters across all HbO time courses in each subject using a finite-impulse response filter (order=1000). Given, frequency dependent contributions associated with resting-state-functional connectivity, we explored intrinsic neural oscillations within the 0.01 - 0.1 Hz low-frequency band. The filtered HbO time series of every channel was correlated with the remaining 15 channels across the entire 6-minute acquisition period using Pearson’s r correlation method, to form a 16 x 16 correlation matrix per subject​. The 16 x 16 correlation matrix of every subject was converted to z-scores using Fisher r to z transformation and an independent sample t-test was performed on the z-scores of every channel pair to identify significant differences in connectivity between low-pain and high-pain groups. FDR-based thresholding was performed at α = 0.05 to account for multiple comparisons. Additionally, the Pearson's r connectivity measures of channel pairs that were statistically different between the two groups were extracted and correlated with self-reported pain scores to examine the relationship between pain levels and functional connectivity strength.

Results: An exploratory analyses of resting-state functional connectivity revealed an overall decrease in connectivity strength between prefrontal and somatosensory cortices in the high pain FOP patients. (**Fig. Supplemental 2A-C**). Functional connectivity results specific to low frequency oscillations within the 0.01 - 0.1 Hz frequency range are shown. A significant (FDR-p_corr_ < 0.0005) decrease in functional connectivity between the prefrontal cortex (C6) and somatosensory cortices (C12) was evident. At the p < 0.01, uncorrected threshold level, a parallel loss of coherence among two other channel pairs (C7-C12 and C8-C10) was observed (**Supplemental Fig. 2D**). A reduction in functional connectivity strength between prefrontal and somatosensory regions is in accord with frequency analysis results, where perturbations within a sub-band (slow-5) that facilitates long-range functional interactions was identified. Additionally, a greater loss in functional connectivity between prefrontal and somatosensory cortices was significantly associated with a higher levels of patient-reported pain intensities. This negative correlation was observed for C6-C12 (r = -0.71, p = 0.021), C7-C12 (r = -0.69, p = 0.027), and C8-C10 (r = -0.64, p = 0.046). Taken together, higher levels of pain in FOP patients are associated with disruption(s) in large-scale networks, where the prefrontal cortex may act as a key dysfunctional CNS hub.


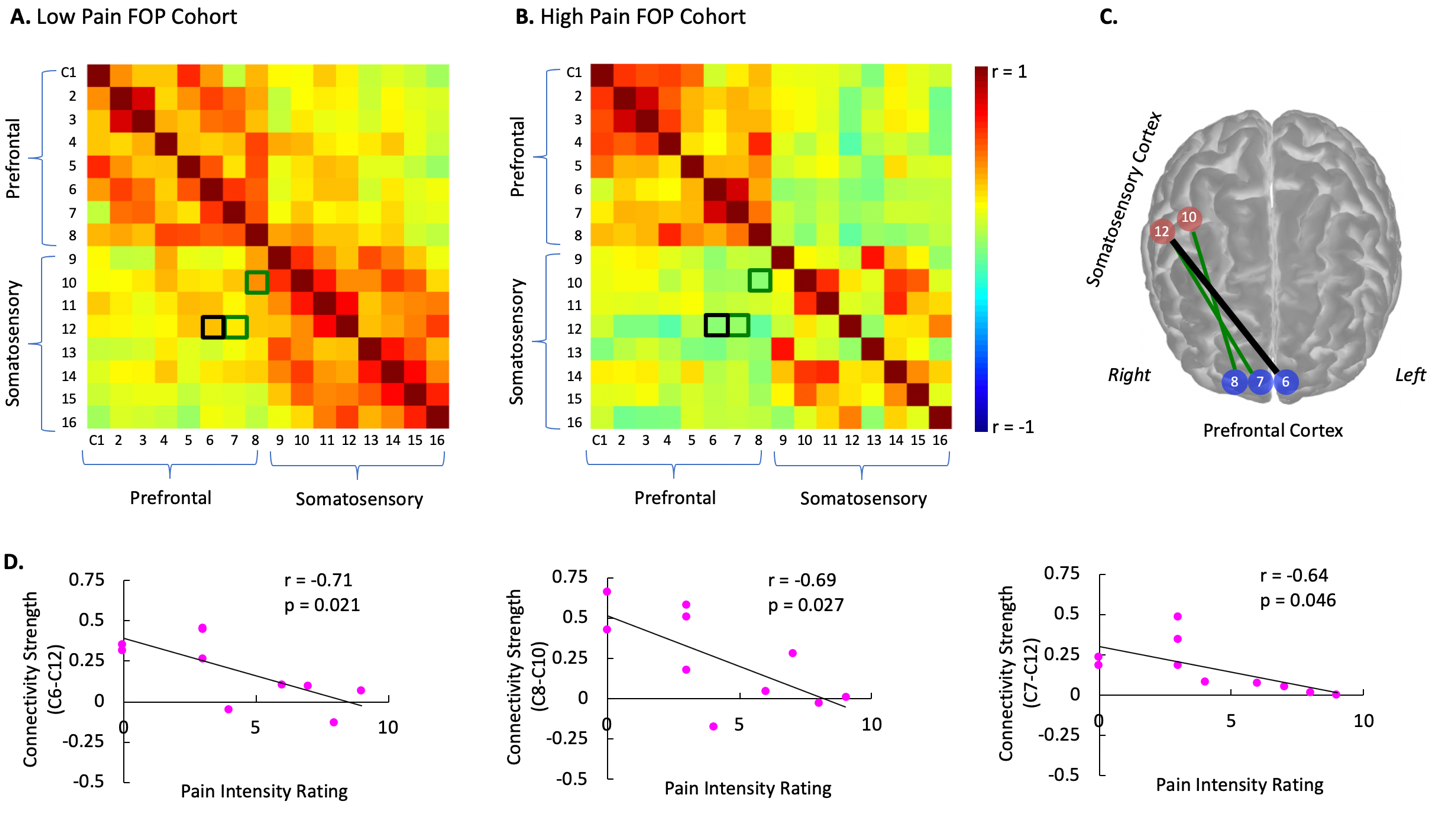


**Supplemental Figure 2: Reduced cortical functional connectivity in FOP patients with high self-reported clinical pain states.** Relative to the low (N=5) pain FOP sub-population (**A.**), high (N=5) pain patients (**B.**) largely showed a decrease in cortical functional connectivity. A channel pair (black boxes) involving the medial prefrontal cortex (C6) and somatosensory cortex (C12) was significantly (FDR-p_corr_ < 0.0005) different between the two FOP sub-populations. At the p_uncorr_ < 0.01 level, two additional pairs (green boxes) also showed decreased connectivity strength in the high pain FOP cohort. (**C.**) Prefrontal cortex and somatosensory cortex channel pairs showing significant differences between low and high pain FOP cohorts are depicted on the brain surface. **D.** A consistent negative correlation between the prefrontal and somatosensory cortex functional connectivity strengths and clinical pain levels was observed.
